# Water-sensitive photoacoustic temperature characterization at 960 nm in cerebral vascular phantoms with CT co-registration

**DOI:** 10.1016/j.pacs.2026.100862

**Published:** 2026-07-21

**Authors:** Chengpeng Chai, Kaiyu Wang, Linke Chen, Jinling Zhang, Yufei Mao, Xiaodi Ren, Zhengyang Li, Feiyan Jin, Yun-Hsuan Chen, Liang Lei, Mohamad Sawan

**Affiliations:** a CenBRAIN Neurotech, School of Engineering, Westlake University, 600 Dunyu Road, Xihu District, Hangzhou, Zhejiang 310030, China; b Institute of Advanced Technology, Westlake Institute for Advanced Study, 18 Shilongshan Street, Xihu District, Hangzhou, Zhejiang 310024, China; c College of Environmental and Resource Sciences, Zhejiang University, 866 Yuhang tang Road, Hangzhou, Zhejiang Province 310058, China; d Key Laboratory of Coastal Environment and Resources of Zhejiang Province, School of Engineering, 18 Shilongshan Road, Hangzhou, Zhejiang Province 310024, China; e School of Medical and Technology, Beijing Institute of Technology, Beijing, China

**Keywords:** Photoacoustic thermometry, Cerebral Vascular Phantoms, Hemoglobin, 960 nm, CT Co-registration, Temperature-bin Analysis, Phantom-based Temperature Characterization

## Abstract

Precise temperature monitoring is important for thermal therapy, vascular physiology, and brain function research. Photoacoustic thermometry often relies on hemoglobin-related wavelengths and is easily affected by hemoglobin concentration, oxygenation, and optical fluence. Here, we propose and validate a water-sensitive photoacoustic thermometry strategy at 960 nm in cerebrovascular phantom configurations containing a straight human whole-blood channel, with and without a bone-like shell. CT-guided registration enabled structurally constrained ROI analysis. Although frame-by-frame PAI signals fluctuated, clear temperature-dependent trends were preserved and strengthened by statistical aggregation. In the phantom without the bone-like shell, 0.1°C temperature-binned median aggregation yielded an apparent calibration R^2^ of 0.996 and a temperature inversion RMSE of 0.08°C; after 100-frame averaging, the noise-equivalent temperature difference decreased to 0.124°C. For the phantom with a bone-like shell, an R^2^ > 0.976 and an RMSE of 0.13°C were still achieved, supporting robust 960 nm photoacoustic thermometry in hemoglobin-containing cerebrovascular phantom environments.

## 1 Introduction

Precise temperature monitoring is a key requirement in numerous biomedical applications. Photoacoustic imaging (PAI) combines the advantages of optical absorption contrast and ultrasound detection depth, enabling high spatial resolution in relatively deep tissues [1], [2], [3], [4]. Meanwhile, the amplitude of photoacoustic signals is inherently affected by temperature, providing a physical basis for the development of noninvasive temperature imaging techniques. Existing studies have shown that the initial photoacoustic pressure is jointly related to the Grüneisen parameter [5], [6] and the optical absorption coefficient [7]. Therefore, when temperature changes the thermal or optical properties of tissue, the photoacoustic signal also changes accordingly, thereby providing a theoretical foundation for photoacoustic thermometry.

In tumor thermal ablation, photothermal therapy, and focused ultrasound treatment, real-time temperature feedback with spatial resolution is an important prerequisite for precisely controlling treatment boundaries and avoiding damage to normal tissues [8], [9], [10]. In neuroscience research, local temperature changes are closely related to cerebral blood flow, metabolic level, and neural activity [5], and photoacoustic thermometry with structural correspondence also has the potential to provide a new label-free characterization method for brain function research [11], [12], [13], and multidimensional optical imaging approaches have further enabled dynamic characterization of neurovascular structure and function [14]. In addition, in vascular physiology and inflammation research, tissue temperature changes can directly reflect local metabolic levels [15], inflammatory responses, and blood perfusion status. Photoacoustic thermometry can simultaneously acquire vascular structural information and temperature distribution imaging, providing multidimensional key information for analyzing related physiological and pathological processes [16].

Although photoacoustic thermometry has clear potential, most existing methods in biomedical scenarios still mainly rely on hemoglobin-related wavelengths, estimating temperature through photoacoustic amplitude changes caused by hemoglobin absorption [17]. However, this approach has three inherent limitations. First, conventional models often attribute the temperature response mainly to changes in the Grüneisen parameter while neglecting the temperature dependence of the absorption coefficient itself, and this assumption is not robust in complex tissues and blood-containing environments [18], [19]. Second, hemoglobin-related signals are highly affected by concentration, oxygenation status, and local optical fluence, all of which exhibit significant spatiotemporal heterogeneity in vivo and are difficult to fully decouple from pure temperature effects [20]. Third, within commonly used wavelength ranges, the temperature response amplitude of hemoglobin absorption is limited, resulting in restricted thermometric sensitivity and robustness, which is particularly unfavorable for high-precision temperature inversion [21]. Although multi-wavelength spectral inversion can compensate for oxygenation interference to some extent, it places higher demands on system complexity, calibration accuracy, and signal-to-noise ratio, and still faces considerable challenges for in vivo applications [17]. Therefore, conventional hemoglobin-based photoacoustic thermometry has inherent limitations in model assumptions, signal stability, and temperature sensitivity. Although representative studies have reported sub-degree performance in controlled media, such as ink (0.15°C [22]), chicken breast tissue (0.6°C [23]), Indian ink (0.3°C [24]), agarose phantoms containing gold nanoparticles under dual-wavelength excitation (0.3°C [25]), and homogeneous porcine blood clots (0.1°C [26]), these results remain strongly dependent on the imaging target, contrast mechanism, and experimental conditions, and there is an urgent need to develop new temperature-sensitive contrast mechanisms and inversion frameworks [27], [28].

To overcome the limitations of hemoglobin-dominated strategies, this study proposes a photoacoustic thermometry approach that uses water rather than hemoglobin as the primary temperature-sensitive target. Water is the most abundant component in soft tissues and has a relatively stable spatial distribution. Its near-infrared absorption characteristics exhibit a predictable response to temperature changes at specific wavelength bands [29], [30], [31]. Around 960 nm, due to the thermally induced restructuring of the hydrogen-bond network between water molecules [29], the probability of molecular vibrational transitions increases significantly with rising temperature [30], [31], thereby producing a characteristic temperature-dependent absorption band of water. Moreover, at this wavelength, the absorption difference between oxyhemoglobin and deoxyhemoglobin is relatively small. Therefore, the sensitivity of the photoacoustic signal to changes in hemoglobin oxygenation status and concentration is relatively reduced, while the response to changes in water absorption becomes relatively prominent [28], [32]. Based on this spectral characteristic, a quantitative thermometric framework centered on the temperature dependence of the water absorption coefficient can be established, thereby improving the robustness of temperature measurement in hemoglobin-containing environments.

Based on the above concept and informed by prior transcranial simulation studies [33], [34], [35], [36], this study constructed human whole blood channel cerebrovascular phantoms without and with a bone-like shell, and combined CT imaging to obtain structural ground truth and achieve cross-modal spatial registration, systematically evaluating the feasibility of 960 nm water- sensitive photoacoustic thermometry in a hemoglobin-containing brain-like environment. Compared with our previous homogeneous-medium study, the present work extends 960 nm temperature characterization to human whole-blood vascular phantoms within the 35–40°C range, introduces CT-guided ROI localization and reconstruction calibration, and evaluates the response with and without a simplified high-sound-speed shell boundary. Through this design, the study aims to assess the feasibility and limitations of 960 nm water-sensitive photoacoustic thermometry in a more complex brain-like phantom environment.

## 2 Materials and methods

### 2.1 Photoacoustic imaging system and wavelength selection

This study used a laboratory-built photoacoustic computed tomography (PACT) system for data acquisition, and its system configuration was consistent with our previous work [37]. The excitation source of the system was a Q-switched Nd: YAG laser (Nimma 900, Beamtech Inc., Beijing), with a fundamental output of 1064 nm. After frequency doubling, a 532 nm pump beam was generated, which further drove an OPO (BB-OPO−532, Deyang Tech Inc., Zhejiang) to output tunable wavelengths from 680 to 980 nm. The laser pulse width was approximately 8.5 ns, with a repetition rate of 10 Hz. The tuned laser was delivered to the sample through a custom fiber bundle (Beijing Reful Co., Ltd., Beijing, China). which consisted of 976 fibers in total, with a total length of 1.6 m and a branch-end size of 30 mm × 0.5 mm, enabling multi-angle illumination of the phantom. The total per-pulse laser energy incident on the phantom surface was approximately 35 mJ. Based on an effective illuminated area of approximately 32 cm^2^ at the phantom surface, the estimated surface optical fluence was approximately 1.1 mJ/cm^2^. This value is below the ANSI maximum permissible exposure level for nanosecond pulsed laser exposure at 960 nm and is within the range where the photoacoustic response can be considered approximately linear with respect to optical absorption.

Because the electrical polarity of the detected PA waveform depends on the transducer and acquisition chain, PA amplitude was defined using a polarity-independent RMS amplitude rather than the signed reconstructed value. For the vascular-phantom experiment, the PA amplitude was quantified using the RMS value within the registered ROI. The complete amplitude sequence was then normalized using min-max normalization.

The photoacoustic signals were received by a custom 128-element semi-ring ultrasound transducer array with a diameter of 110 mm and a center frequency of 5 MHz. The signals were acquired by a 128-channel data acquisition system (Marsonics128, Langyuan Tech. Inc., Tianjin) at a sampling rate of 80 MSPS, thereby balancing imaging depth and spatial resolution.

In this study, 960 nm was selected as the primary thermometry wavelength. The reasons for choosing this wavelength are as follows: first, water has a distinct absorption band near this wavelength, and its absorption coefficient changes monotonically with increasing temperature; second, temperature-dependent NIR measurements of whole human blood have identified a detectable water-related response around 960 nm, representing the most significant temperature-dependent spectral effect between 800 and 1000 nm [38], which helps reduce interference caused by changes in oxygenation state; third, our preliminary homogeneous medium experiments showed that under the 960 nm condition, a good linear relationship can be established between the water-related photoacoustic signal and temperature [7]. Although an isosbestic point at approximately 1003.4 nm was identified from the spectrophotometric measurements, it could not be evaluated in the present PA experiment because it was outside the available OPO wavelength range.

### 2.2 Brain-mimicking agarose phantom fabrication

To construct a cerebrovascular phantom suitable for multimodal registration, this study employed an agarose casting method combined with post-embedded blood channels. First, agarose powder was dissolved in ultrapure water at a ratio of 1 g/100 mL, heated until fully transparent, cooled to below 38°C, and then poured into a high-temperature-resistant 8001 SLA photosensitive resin 3D-printed mold with dimensions of 3.5 cm × 3.5 cm × 5 cm.

The vascular channel was constructed using high-temperature-resistant polytetra-fluoroethylene (PTFE) tubing, with an inner diameter of 0.8 mm and an outer diameter of 1.6 mm. The tubing was pre-filled with human whole blood and sealed using silicone blind-hole plugs combined with photocurable adhesive to prevent leakage of the perfusate. The tubing was then fixed at the center of the mold so that the vascular channel was located in the central region of the phantom. After the agarose had fully solidified, a phantom structure with cerebrovascular geometric characteristics was formed.

Considering that the ultrasound speed of cured SLA resin materials can usually reach approximately the order of 3.0 km/s, which is significantly higher than that of the hydrogel background and liquid coupling medium, the 8001 SLA shell can serve as a bone-like high-sound-speed boundary to introduce skull-like acoustic mismatch effects. The estimated acoustic properties of the agarose background and SLA shell, together with representative values for human brain parenchyma and skull, are summarized in Table S4 of the Supplementary Material S2. It should be noted that this shell does not fully reproduce the multilayered structure, attenuation, dispersion, anisotropy, or morphological heterogeneity of the human skull. This design takes into account both the blood-containing absorption channel required for photoacoustic imaging and the stable geometric boundary required for CT imaging, which is conducive to subsequent multimodal image registration and temperature-related photoacoustic response analysis. Human whole blood samples were obtained from healthy volunteers, and collection and use were completed in accordance with institutional ethical requirements. All participants signed written informed consent forms.

### 2.3 CT imaging and multimodal registration

**Fig. 1:**
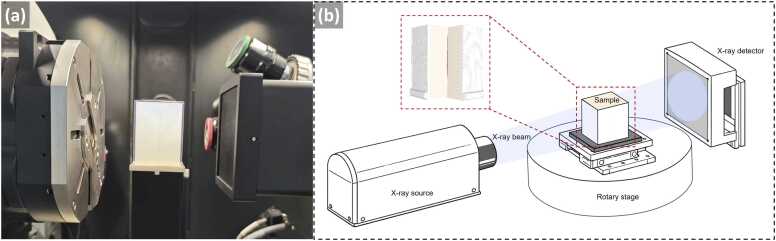
CT imaging experiment of the phantom and its structural schematic. (a) Photograph of the phantom placed in the CT system; (b) schematic diagram of the CT imaging setup composed of the X-ray source, rotation stage, and detector.

To obtain a reference standard for the vascular structure of the phantom and achieve spatial correspondence with the photoacoustic images, X-ray computed tomography (CT) was used for structural imaging. CT scanning was performed using a Zeiss Xradia 610 Versa system with scanning parameters of 80 kV, 100 μA, and 1 s exposure per frame, and a total of 1018 projection images were acquired. The reconstructed voxel size was approximately 35.390 μm, which was sufficient for high-resolution visualization of the vascular channel structure. The raw image sequence was initially processed and converted in ImageJ, then segmented in ilastik based on intensity, texture, and shape features, and further used for three-dimensional registration, as shown in Fig. 1.

It should be noted that CT scan was acquired after completion of the PA temperature experiment and was used only to obtain structural ground truth for subsequent CT-PA registration and ROI localization. Therefore, possible X-ray-induced heating or blood-property changes during CT acquisition did not affect the PA temperature measurements reported in this study.

The registration between the photoacoustic images and the CT model adopted a rigid registration strategy. In 3D Slicer, visible edge landmarks and structural boundaries shared by the CT image and PA reconstruction were used as shared spatial markers to establish a unified coordinate system. To further correct geometric scale deviations in the photoacoustic images, the known geometric dimensions of the phantom shell were used for scale correction of the reconstructed results, and sound speed correction was completed in combination with the known structural spacing. After registration and correction, the vascular structures extracted from CT could be mapped onto the photoacoustic images to guide the precise selection of the ROI, thereby providing a reliable anatomical basis for subsequent quantitative analysis.

### 2.4 Temperature reference measurement and photoacoustic thermometry model

**Fig. 2:**
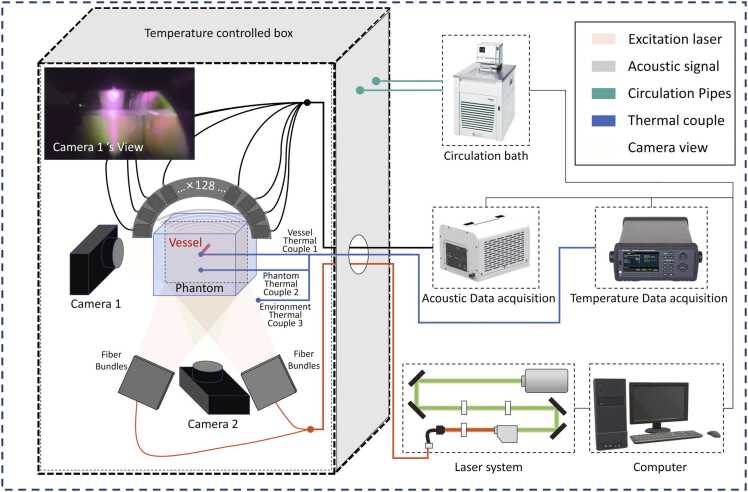
Schematic of the photoacoustic temperature measurement system with an inset camera-view photograph of the experimental setup inside the temperature-controlled box. The system consists of a temperature-controlled box, an excitation laser module, line-shaped optical illumination, a single 128-element semi-ring ultrasound transducer array, a data acquisition unit, a temperature recording module, and a host computer for control and processing. The sample was illuminated by the pulsed excitation laser, and the generated photoacoustic signals were detected by the semi-ring ultrasound array and transferred to the acquisition system for image reconstruction and temperature-related analysis. Colored lines indicate the functional connections among the temperature-control, laser-excitation, acoustic-detection, signal-acquisition, and data-processing modules.

A multi-stage circulating fluid temperature control system was used in the experiment to establish a stable thermal field. The phantom was placed in a double-layer temperature-controlled chamber, where uniform heat exchange was achieved through circulating fluid, as shown in Fig. 2. A photograph of the experimental setup and a detailed schematic layout are provided in Supplementary Material S1. During the experiment, the peripheral water bath circulation was controlled to linearly increase/decrease between 35°C and 40°C within 1 h, and after the heating process was completed, it was maintained for 6 h to achieve uniform temperature control of the phantom by means of the heat capacity inside the device, so as to ensure thermal equilibrium. During the heating and cooling periods, the photoacoustic system continuously recorded data. Temperature was monitored using three T-type thermocouples positioned adjacent to the PTFE blood channel, within the agarose phantom, and in the surrounding dimethyl silicone-oil bath, respectively, without obstructing the photoacoustic optical path. The thermocouples were subjected to two-point correction using an ice-water mixture and boiling water, and the stated ±0.02°C represents the nominal instrument resolution rather than independently calibrated measurement accuracy. Because no thermocouple was inserted into the blood channel, the recorded temperatures represent local temperatures surrounding the vessel rather than direct intraluminal blood temperature. The phantom was placed on a three-axis adjustable sample holder to achieve precise spatial positioning. In addition, the system was equipped with dual cameras (DJI Nano & Action 5 Pro, DJI, Shenzhen, China) to record the experimental process in real time, for auxiliary monitoring of the experimental status and result verification.

The initial photoacoustic pressure amplitude satisfies:

(1) $$p_{0}\left(\lambda_{i}\right)=\Gamma_{n}\eta_{\mathrm{th}}\mu_{a}\left(\lambda_{i}\right)F_{\mathrm{pulse}}$$

where $\Gamma_{n}$ is the Grüneisen parameter, $\mu_{a}\left(\lambda_{i}\right)$ is the absorption coefficient, and $F_{\mathrm{pulse}}$ is the local optical fluence. In traditional photoacoustic thermometry models, the temperature dependence is mainly attributed to changes in $\Gamma_{n}$; however, under controlled experimental conditions, the temperature dependence of the ideal initial PA pressure may arise jointly from the temperature-dependent Grüneisen parameter and optical absorption coefficient $\mu_{a}\left(\lambda_{i}\right)$ [5], [7].

Structure was first selected based on the CT registration results, and the photoacoustic signal within this region was extracted for quantitative analysis. To achieve accurate matching between temperature records and photoacoustic images, time alignment was completed according to the real acquisition time recorded in the file name of each reconstructed image frame, and the photoacoustic data were linearly interpolated and registered with the temperature sensor recordings. Subsequently, the root mean square (RMS) amplitude of the photoacoustic signal within the ROI was used as the main analysis metric, and its relationship with temperature was analyzed at three statistical levels: frame-by-frame, fixed-frame averaging, and 0.1°C temperature binning. A bin width of 0.1°C was selected as a predefined statistical aggregation window based on the temperature-control resolution, sampling density, and frame-to-frame PA fluctuation level. The purpose of this binning strategy was to reduce the influence of frame-level fluctuations and evaluate the stability of the temperature-dependent PA response after aggregation. For the temperature-binning analysis, the median value within each temperature bin was used as the representative value, and linear fitting was performed separately for data at different statistical levels. The coefficient of determination (R^2^) was used to evaluate the linearity of the temperature-related photoacoustic response.

### 2.5 Evaluation metrics

To quantitatively evaluate temperature inversion performance, this study used the coefficient of determination (R^2^) to evaluate the linearity of the temperature-photoacoustic signal relationship, and used the root mean square error (RMSE) to evaluate the apparent calibration error:

(2) $$R^{2}=1-\frac{\sum_{i=1}^{n}{(T_{i}-{\hat{T}}_{i})}^{2}}{\sum_{i=1}^{n}{(T_{i}-\overset{ˉ}{T})}^{2}}$$

(3) $$\mathrm{RMSE}=\sqrt{\frac{1}{n}\sum_{i=1}^{n}{\left(T_{i,\mathrm{pred}}|,T_{i,\mathrm{true}}\right)}^{2}}$$

where $T_{i}$ is the reference temperature, and ${\hat{T}}_{i}$ is the estimated temperature. In addition, the noise-equivalent temperature difference (NEΔT) was calculated to characterize the frame-level thermometric sensitivity. NEΔT was defined as the standard deviation of the residual PA signal divided by the fitted PA-temperature sensitivity:

(4) $$\mathrm{NE}\Delta T=\sigma_{\mathrm{res}}/|\mathrm{dS}/\mathrm{dT}|$$

where Srepresents the ROI-based RMS PA amplitude, dS/dTis the slope of the linear PA-temperature fitting, and $\sigma_{\mathrm{res}}$is the standard deviation of the residual PA signal. All metrics were calculated within the ROI determined after CT registration. NEΔT was reported for raw frame-wise data and 100-frame averaging to distinguish frame-level noise-equivalent sensitivity from the apparent temperature inversion error obtained after temperature-binned aggregation.

## 3 Results

### 3.1 Wavelength comparison in homogeneous media

**Fig. 3:**
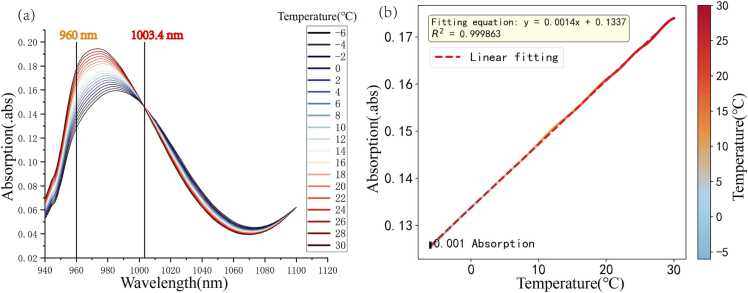
Temperature-dependent absorption characteristics of ultrapure water. (a) Absorption spectra of ultrapure water in the 940–1120 nm band at different temperatures; (b) Relationship between absorbance and temperature at 960 nm. The relevant results have been published in our previous work [7] and are used here as the basis for wavelength selection in the phantom experiments of this extended study.

To verify the physical basis of the 960 nm water-sensitive photoacoustic thermometry mechanism, the temperature-dependent variation of absorption characteristics in the near-infrared band was first analyzed in homogeneous water samples. The results showed that, near 960 nm, the absorption coefficient of water increased monotonically with increasing temperature and exhibited an excellent linear relationship (R^2^ = 0.999863). As the temperature increased, the absorption spectrum as a whole shifted toward longer wavelengths, resulting in enhanced absorption at a fixed wavelength. This result indicates that 960 nm can provide a stable and predictable temperature response and is an appropriate wavelength for constructing a water-sensitive photoacoustic thermometry model, as shown in Fig. 3.

**Fig. 4:**
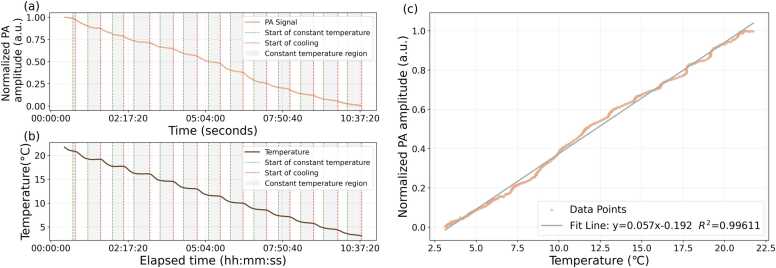
Experimental results of the relationship between photoacoustic signal and temperature in a homogeneous medium. (a) Photoacoustic signal as a function of time; (b) Temperature signal as a function of time; (c) Relationship between photoacoustic signal and temperature. The relevant results have been published in our previous work [7].

Furthermore, pure water photoacoustic signals corresponding to different temperatures were acquired under the 960 nm condition, and it was found that the photoacoustic signal intensity also had a highly linear relationship with temperature (R^2^ = 0.9961), as shown in Fig. 4. This trend was consistent with the temperature-dependent absorption spectra, indicating that the change in water absorption was reflected in the PA response under the controlled homogeneous-medium conditions. However, the measured PA response may also include contributions from the temperature dependence of the Grüneisen parameter and other system-related factors. This phenomenon provides a mechanistic basis for the subsequent experiments in hemoglobin-containing phantoms.

In summary, the homogeneous-medium experiments shown in Fig. 3, Fig. 4, which were previously reported in our EMBC 2025 study, provided the prior experimental basis for selecting 960 nm from both the optical absorption and photoacoustic signal perspectives. However, the lower-temperature calibration relationship obtained from these experiments was not extrapolated to the vascular-phantom study. Instead, the PA amplitude-temperature relationship in the whole-blood phantom was independently characterized using data acquired within the 35–40°C range.

### 3.2 Spatial validation and cross-modal registration guided by CT results

**Fig. 5:**
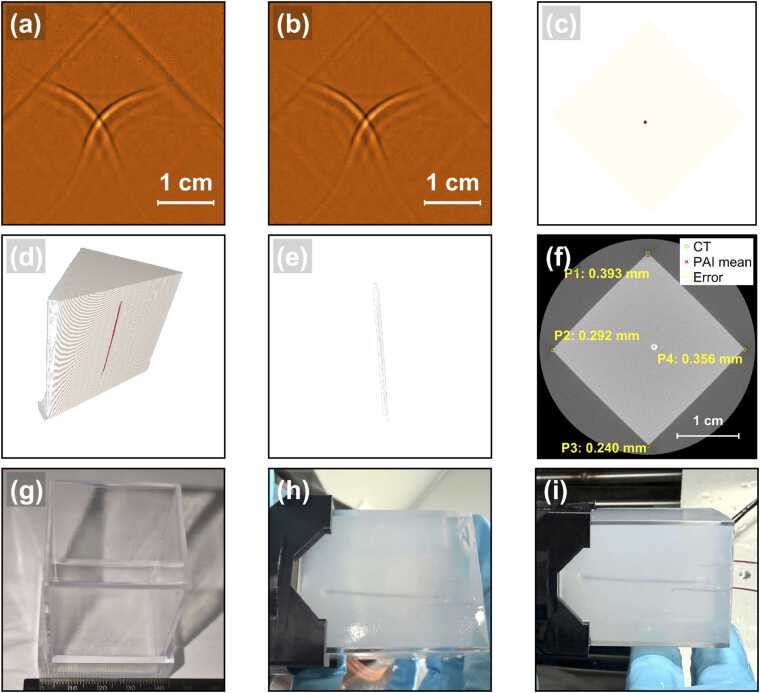
CT-guided spatial validation and cross-modal registration in the bone-like shell phantom. (a) DAS photoacoustic reconstruction; (b) BP photoacoustic reconstruction. (c) CT-derived structure of the blood channel and PTFE tubing; (d) Longitudinal CT slice of the phantom; (e) Transverse CT slice of the phantom, Supplementary Material S3 and S4 provides a rotating visualization of the three-dimensional structure. (f) CT-PA landmark registration overlay showing CT landmarks, mean registered PA landmarks, and registration error vectors; (g) Photograph of the bone-like shell phantom with a scale bar; (h, i) Photographs of the phantom before and after the heating cycle, respectively, taken from the same view to show the macroscopic state of the phantom and the position of the thermal couple.

To verify the spatial reliability of photoacoustic thermometry analysis, this study further conducted cross-modal registration of CT and photoacoustic images in a cerebrovascular phantom. Using the corner points of the phantom shell and known geometric structures as spatial references, rigid registration was completed in 3D Slicer, and scale of the photoacoustic images and the sound speed variation caused by temperature changes were further corrected using structures with known spacing. After correction, the external phantom boundary in the photoacoustic reconstructed image corresponded well with the structural contour extracted from CT, indicating that the established registration and scale correction workflow can effectively constrain the spatial position of the photoacoustic image, as shown in Fig. 5. And 20 PA frames around 37.5°C were selected as representative mid-temperature reconstructions, and the reconstruction sound speed was iteratively tested from 900 to 1200 m/s. A fixed sound speed of 1095 m/s was selected because it provided the best spatial consistency between the PA reconstruction and the CT-derived structure. The CT-PA registration accuracy was quantitatively evaluated using 10 representative PA reconstructions and 40 corner landmarks. The overall mean target registration error (TRE) was 0.3204 mm, with an overall TRE RMSE 0.3491 mm and a maximum TRE of 0.5923 mm. The mean TRE of individual PA reconstructions ranged from 0.1158 to 0.4928 mm. These results indicate that CT-guided registration provided sub-millimeter-level spatial correspondence for subsequent ROI localization. The registration error shown in Fig. 5(f).

In the registered photoacoustic images, the outer boundary of the phantom frame was clearly displayed, and the internal vascular channel could also be clearly identified. However, an obvious X-shaped crossed signal distribution could be observed around the main vascular structure. Comparison between DAS and BP reconstruction results showed that this X-shaped structure existed in both reconstruction methods, but was more prominent in the BP images, suggesting that it was more likely to originate from reconstruction artifacts caused by strong boundary back-projection under limited-view conditions rather than the true vascular geometry itself. Specifically, as a slender high-contrast target, the upper and lower boundaries of the vessel, as well as the strong contrast between the vessel and the external shell, may both generate crossed streak artifacts under semi-ring array limited-aperture imaging conditions; the sound speed mismatch of the Teflon outer wall of the vascular tube may further enhance this phenomenon. Nevertheless, this artifact did not destroy the distinguishability of the main vascular contour, and the ROI could still be stably selected under CT structural constraints, thereby ensuring the spatial consistency of subsequent temperature-related signal analysis.

### 3.3 Temperature-dependent photoacoustic response in a hemoglobin-containing cerebrovascular phantoms

**Fig. 6:**
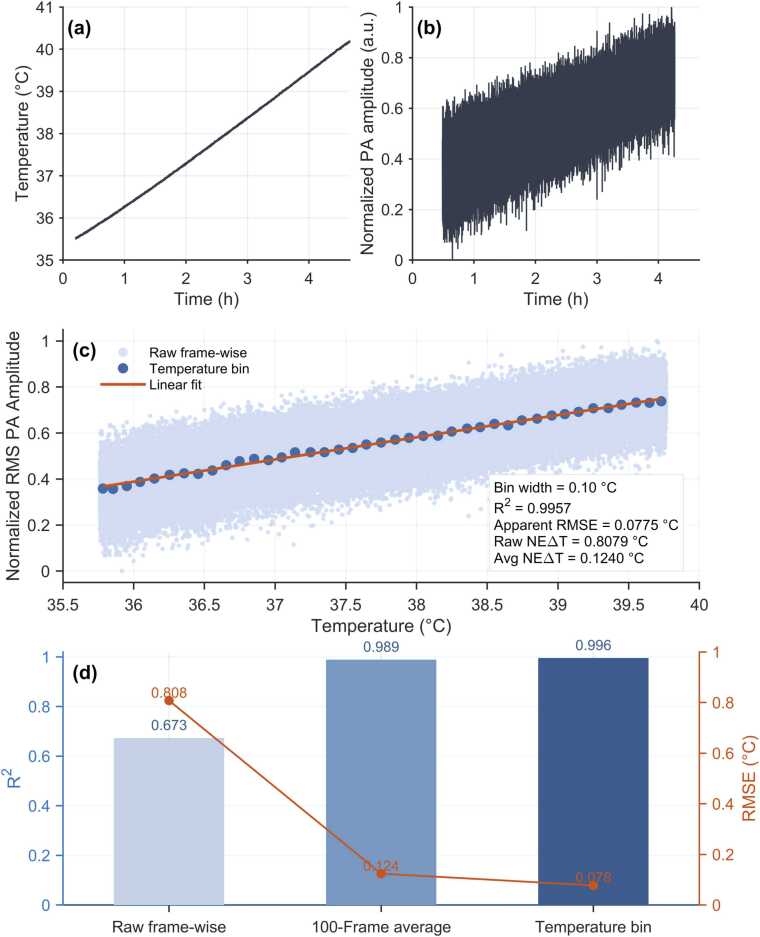
Quantitative results of the temperature-related photoacoustic response in a cerebrovascular phantom without a bone-like shell. (a) Variation of the reference temperature over time during heating; (b) Variation of RMS photoacoustic amplitude within the selected ROI over time; (c) Relationship between temperature and ROI-based RMS photoacoustic amplitude, showing raw frame-wise data, 0.10°C temperature-binned median values, and the corresponding linear fit; the annotated NEΔT values represent the noise-equivalent temperature differences for raw frame-wise and 100-frame averaged analyses; (d) Comparison of R2 and apparent temperature inversion error (RMSE) under three statistical strategies: raw frame-by-frame, frame averaging, and temperature binning.

After completing CT-guided ROI selection, the relationship between the vascular photoacoustic signal within the ROI and temperature under the 960 nm condition was further analyzed. Based on the alignment results between the actual acquisition time and the temperature records, the RMS amplitude within the ROI was extracted from the frame-by-frame reconstructed images, and the temperature-photoacoustic intensity relationship was evaluated at three statistical levels: raw frame-by-frame data, 100-frame averaging, and 0.1°C temperature binning. The main figures in the main text use the median of the 0.1°C temperature bins as the primary presentation method, as shown in Fig. 6.

The results showed that, in the hemoglobin-containing cerebrovascular phantom, the photoacoustic response within the ROI exhibited an overall clear temperature-dependent trend as the temperature increased. Although the raw frame-by-frame data showed obvious fluctuations, a stable monotonic change could still be observed from the scatter distribution. Compared with the raw frame-by-frame results, the temperature-photoacoustic intensity relationship was significantly enhanced after statistical aggregation, among which the 0.1°C temperature binning results achieved the best linear characterization performance. For the phantom without a bone-like shell, the raw frame-wise analysis yielded $R^{2}=0.673$, reflecting considerable frame-level PA fluctuations. After 100-frame averaging, $R^{2}$increased to 0.989 and NEΔT decreased from 0.8079°C to 0.1240°C, indicating that a substantial component of the frame-wise variation could be reduced through temporal averaging. The 0.1°C temperature-binned median analysis was then used to further characterize the underlying temperature-dependent PA response after statistical aggregation, yielding $R^{2}=0.9957$and an apparent temperature inversion RMSE of 0.0773°C.

Since the phantom with a bone-like shell and the phantom without a shell showed consistent overall temperature-related variation trends, and the main difference was only a slight decrease in quantitative metrics, the main figures in the text present only the representative results of the phantom without a bone-like shell. For the phantom with a bone-like shell, the temperature-photoacoustic intensity relationship still maintained a high linearity, reaching R^2^ > 0.976, with a corresponding apparent temperature inversion error of RMSE = 0.13°C, indicating that the temperature-dependent PA response remained consistently observable after introducing the simplified high-sound-speed boundary.

## 4 Discussion

This study verified the feasibility of a water-sensitive photoacoustic thermometry strategy based on the 960 nm wavelength in a hemoglobin-containing cerebrovascular phantom. Homogeneous medium experiments showed that both the absorption of water and the corresponding photoacoustic signals at 960 nm had a highly linear relationship with temperature; in the cerebrovascular phantom, the RMS photoacoustic amplitude within the ROI extracted after CT registration also exhibited a clear temperature correlation. This indicates that, in a complex blood-containing environment, the photoacoustic response at 960 nm can still provide stable temperature-related information.

Compared with traditional thermometry approaches that rely on changes in hemoglobin absorption, the advantage of the 960 nm strategy lies in the fact that its temperature-sensitive mechanism depends more on changes in the absorption coefficient of water, thereby reducing interference from hemoglobin concentration, oxygenation state, and local perfusion differences in temperature inversion. However, because the vascular phantom contained whole blood and only a single wavelength was used, the present results do not independently determine the relative contributions of water, Hb/HbO_2_, optical fluence, and other system-dependent factors.

CT co-registration provided post-experimental structural ground truth for vascular ROI localization, geometric scale correction, and sound-speed adjustment, particularly in the phantom containing the high-sound-speed shell. Because CT was acquired after the PA experiment, the CT-derived structure represents the post-experiment phantom geometry used for registration. Small geometric drift between the PA and CT acquisitions cannot be completely excluded. No obvious macroscopic deformation, leakage, or bubble formation was observed after the heating process, supporting the use of the CT structure as the spatial reference for ROI localization. The X-shaped structure observed around the vessel was present in both DAS and BP reconstructions and was more pronounced in BP, indicating that it most likely originated from limited-view and boundary-related reconstruction artifacts rather than the true vascular geometry. Nevertheless, the main vascular contour remained distinguishable and could still be consistently localized using CT guidance.

From the data analysis results, although the raw frame-by-frame data exhibited relatively large fluctuations, the overall data still showed a clear temperature-related linear trend; after statistical aggregation, the temperature-photoacoustic intensity relationship was significantly enhanced, and the 0.1°C temperature binning results outperformed fixed-frame averaging. Although the RMSE obtained from the temperature-binned median analysis represents an apparent temperature inversion error after statistical aggregation rather than single-frame thermometric precision, the improved linearity indicates that the dispersion in the current data may not be entirely random, but more likely includes superimposed systematic fluctuations. Pulse-energy fluctuations may be an important source of the remaining frame-wise variability, and future pulse-by-pulse optical-energy normalization may further improve quantitative stability.

This study still has several limitations. First, the experiments were conducted using static phantoms and did not include perfusion, thermal convection, repeated heating-cooling cycles, independently fabricated phantom replication, or held-out prediction. Because static whole blood was used over an extended experimental period, gradual changes in blood state cannot be completely excluded as a potential time-correlated confound. Hematocrit, metHb, SO_2_, and paired pre/post spectrophotometric measurements were not obtained in the present experiment. Moreover, the calibration derived from the present phantom should not be directly transferred to in vivo absolute thermometry, where absorber concentration, optical fluence, and tissue properties may vary spatially and direct temperature ground truth may not be readily available. The present results should therefore be interpreted as controlled phantom-level feasibility validation rather than a universal in vivo calibration.

Second, the current analysis is mainly based on ROI statistics rather than pixel-level temperature field reconstruction. Third, the agarose matrix and SLA shell were not designed to fully reproduce the optical, thermal, and acoustic properties of brain tissue and skull. In particular, the SLA shell did not reproduce the layered, porous, and curved skull structure, frequency-dependent attenuation, anisotropy, or longitudinal-to-shear mode conversion. It was used only to introduce a simplified high-sound-speed boundary. Finally, the current system has not yet introduced pulse-by-pulse optical intensity normalization, and therefore there is still room for further improvement in quantitative stability at the single-frame level.

Future work should therefore proceed in a stepwise manner. In particular, the current framework can be extended to perfused flow phantoms with more realistic neurovascular conditions [39], while more representative transcranial acoustic models, such as ex vivo skull specimens or better calibrated skull-mimicking models, should be introduced to evaluate the robustness of the 960 nm strategy under skull-induced distortion and aberration [40], [41], [42]. On this basis, further validation in ex vivo or small-animal imaging settings [39], [43] would help bridge the current structurally guided phantom characterization toward more biologically relevant brain thermometry.

## 5 Conclusion

In this study, we proposed and validated a water-sensitive photoacoustic thermometry strategy based on the 960 nm wavelength. Homogeneous medium experiments showed that the absorption of water and the corresponding photoacoustic signal at 960 nm had a good linear relationship with temperature, providing a basis for using this band as a temperature-sensitive excitation wavelength. In a hemoglobin-containing cerebrovascular phantom, combined with CT-guided cross-modal registration and ROI analysis, the photoacoustic signal still exhibited a clear temperature dependence. For the phantom without a bone-like shell, the median analysis with 0.1°C temperature binning achieved the best linear characterization performance, with a corresponding apparent temperature inversion error of RMSE = 0.08°C; for the phantom with a bone-like shell, RMSE = 0.13°C could still be maintained. Overall, the 960 nm water-sensitive photoacoustic thermometry strategy, combined with CT structural constraints and temperature-binning statistics, demonstrated consistent temperature-related PA characterization across the tested phantom configurations. Further incorporation of pulse-energy normalization may improve quantitative stability and support the development of noninvasive deep thermometry.

## Institutional Review Board Statement

This study was approved by the Institutional Review Board of Westlake University (Approval No. 20260420GCP001).

## Informed Consent Statement

Written informed consent was obtained from the volunteer involved in this study.

## Funding

This research was funded by CAST Youth Science and Technology Talent Cultivation Program for Doctoral Students, Young Research Fellow Grant of the Joint Academy on Future Humanity[2025XH0104], Research fund for international senior scientists (RFISS) [W2431058], Key Research and Development Program of Zhejiang Province ([2023SDXHDX0005],[2024C03040]), "Pioneer" and "Leading Goose" R&D Program of Zhejiang Province [2024C03002], Westlake University [103186021801], and the Key Project of Westlake Institute for Optoelectronics [2023GD004].

## CRediT authorship contribution statement

**Zhengyang Li:** Writing – review & editing, Writing – original draft, Visualization, Validation, Resources, Methodology, Investigation, Formal analysis, Data curation, Conceptualization. **Feiyan Jin:** Writing – original draft, Visualization, Software, Methodology, Formal analysis, Data curation, Conceptualization. **Yun-Hsuan Chen:** Writing – review & editing, Writing – original draft, Visualization, Validation, Supervision, Software, Resources, Project administration, Methodology, Investigation, Funding acquisition, Formal analysis, Data curation, Conceptualization. **Liang Lei:** Writing – review & editing, Writing – original draft, Visualization, Validation, Supervision, Software, Resources, Project administration, Investigation, Funding acquisition, Formal analysis, Conceptualization. **Mohamad Sawan:** Writing – review & editing, Writing – original draft, Visualization, Supervision, Resources, Project administration, Funding acquisition, Conceptualization. **Chengpeng Chai:** Writing – review & editing, Writing – original draft, Visualization, Validation, Software, Resources, Project administration, Methodology, Investigation, Funding acquisition, Formal analysis, Data curation, Conceptualization. **Kaiyu Wang:** Writing – review & editing, Writing – original draft, Visualization, Validation, Software, Project administration, Methodology, Investigation, Formal analysis, Data curation, Conceptualization. **Linke Chen:** Writing – original draft, Visualization, Validation, Software, Project administration, Investigation, Formal analysis, Data curation. **Jinling Zhang:** Writing – original draft, Visualization, Validation, Software, Resources, Methodology, Investigation, Formal analysis, Data curation. **Yufei Mao:** Writing – review & editing, Visualization, Software, Project administration, Methodology, Investigation, Formal analysis, Data curation. **Xiaodi Ren:** Writing – original draft, Visualization, Validation, Software, Project administration, Methodology, Investigation, Formal analysis, Data curation.

## Declaration of Competing Interest

The authors declare the following financial interests/personal relationships which may be considered as potential competing interests: Chengpeng Chai, Kaiyu Wang, Yun-Hsuan Chen, Liang Lei, and Mohamad Sawan are named inventors on six patent filings related to the technology described in this work, comprising four patent families, including two corresponding Chinese and international filings. Two of these patents have been granted. The patent rights and applications are assigned to Westlake University. All other authors declare no known competing financial interests or personal relationships that could have appeared to influence the work reported in this paper.

## Data Availability

Data will be made available on request.

## Appendix A Supplementary material

S1 Experimental setup and schematic layout

S2 Acoustic parameters of the phantom and skull tissue

S3 360-degree rotation of CT-scanned shelled phantom

S4 30-degree rotation quarter-sectioned shelled phantom

## References

[1] Wei X., Gu B. (2021).

[2] Laufer J. (2024). Quantification of Biophysical Parameters in Medical Imaging.

[3] Yang X. (2021). Photoacoustic imaging for monitoring of stroke diseases: A review. Photoacoustics.

[4] Menozzi L., Yao J. (2024). Deep tissue photoacoustic imaging with light and sound. npj Imaging.

[5] Petrova E. (2013). Using optoacoustic imaging for measuring the temperature dependence of Grüneisen parameter in optically absorbing solutions. Opt. Express.

[6] Petrova E. (2017). Temperature-dependent optoacoustic response and transient through zero Grüneisen parameter in optically contrasted media. Photoacoustics.

[7] Chai C. (2025). 2025 47th Annual International Conference of the IEEE Engineering in Medicine and Biology Society (EMBC).

[8] Ma Y. (2023). Multi-wavelength photoacoustic temperature feedback based photothermal therapy method and system. Pharmaceutics.

[9] Wu D. (2025). Real-time, tissue-adaptive photoacoustic thermometry for precision endoscopic thermal therapy. Laser & Photonics Rev..

[10] Wang T. (2026). Photoacoustic microscopy for multiscale biological system visualization and clinical translation. Adv. Sci..

[11] Yao J., Wang L.V. (2014). Photoacoustic brain imaging: from microscopic to macroscopic scales. Neurophotonics.

[12] Chai C. (2025). Multimodal fusion of magnetoencephalography and photoacoustic imaging based on optical pump: trends for wearable and noninvasive Brain–Computer interface. Biosens. Bioelectron..

[13] Na S. (2022). Massively parallel functional photoacoustic computed tomography of the human brain. Nat. Biomed. Eng..

[14] Li H. (2025). Multidimensional dynamic optical imaging unveils anesthetic-driven hemispheric lateralization in blood-brain barrier homeostasis. Sci. Adv..

[15] Liao L.-D. (2013). Imaging of temperature dependent hemodynamics in the rat sciatic nerve by functional photoacoustic microscopy. Biomed. Eng. Online.

[16] Zhang R. (2023). Hybrid photoacoustic ultrasound imaging system for cold-induced vasoconstriction and vasodilation monitoring. IEEE Trans. Biomed. Eng..

[17] Liu C., Wang L. (2022). Functional photoacoustic microscopy of hemodynamics: a review. Biomed. Eng. Lett..

[18] Amiri H., Makkiabadi B. (2020). A review of ultrasound thermometry techniques. Front. Biomed. Technol..

[19] Chen F. (2023). Absolute Grüneisen parameter measurement in deep tissue based on X-ray-induced acoustic computed tomography. Biomed. Opt. Express.

[20] Buyanov D. (2022). An algorithm for measuring absolute and relative hemoglobin concentrations using near infrared spectroscopy. Biomed. Eng..

[21] Askari M. (2021). A near infrared plasmonic perfect absorber as a sensor for hemoglobin concentration detection. Opt. Quantum Electron..

[22] Pramanik M., Wang L.V. (2009). Thermoacoustic and photoacoustic sensing of temperature. J. Biomed. Opt..

[23] Yao J. (2013). Absolute photoacoustic thermometry in deep tissue. Opt. Lett..

[24] Qin Z., Lai P., Sun M. (2024). Photoacoustic thermal-strain measurement towards noninvasive and accurate temperature mapping in photothermal therapy. Photoacoustics.

[25] Meng L. (2019). Photoacoustic temperature imaging based on multi-wavelength excitation. Photoacoustics.

[26] Jian X. (2020). Multiwavelength photoacoustic temperature measurement with phantom and ex-vivo tissue. Opt. Commun..

[27] Liu Y. (2025). Photoacoustic temperature monitoring technology and biomedical applications. J. Innov. Opt. Health Sci..

[28] Hui X., Malik M.O., Pramanik M. (2022). Looking deep inside tissue with photoacoustic molecular probes: a review. J. Biomed. Opt..

[29] Brubach J.-B. (2005). Signatures of the hydrogen bonding in the infrared bands of water. J. Chem. Phys..

[30] Renati P. (2019). Temperature dependence analysis of the NIR spectra of liquid water confirms the existence of two phases, one of which is in a coherent state. J. Mol. Liq..

[31] Zhou J. (2019). Temperature dependent optical and dielectric properties of liquid water studied by terahertz time-domain spectroscopy. Aip Adv..

[32] Pattyn A. (2021). Model-based optical and acoustical compensation for photoacoustic tomography of heterogeneous mediums. Photoacoustics.

[33] Yang X. (2025). Skull impact on photoacoustic imaging of multi-layered brain tissues with embedded blood vessel under different optical source types: Modeling and simulation. Bioengineering.

[34] Yang X. (2024). Optical transmission in single-layer brain tissues under different optical source types: modelling and simulation. Bioengineering.

[35] Kirchner T., Villringer C., Laufer J. (2023). Evaluation of ultrasound sensors for transcranial photoacoustic sensing and imaging. Photoacoustics.

[36] Graham M.T. (2020). Simulations and human cadaver head studies to identify optimal acoustic receiver locations for minimally invasive photoacoustic-guided neurosurgery. Photoacoustics.

[37] Chai C. (2024). Enhancing photoacoustic imaging for lung diagnostics and BCI communication: simulation of cavity structures artifact generation and evaluation of noise reduction techniques. Front. Bioeng. Biotechnol..

[38] Martinsen P. (2008). Temperature dependence of near-infrared spectra of whole blood. J. Biomed. Opt..

[39] Na S., Wang L.V. (2021). Photoacoustic computed tomography for functional human brain imaging. Biomed. Opt. Express.

[40] Liang B. (2021). Acoustic impact of the human skull on transcranial photoacoustic imaging. Biomed. Opt. Express.

[41] Mao Q. (2024). Ultrasound-assisted aberration correction of transcranial photoacoustic imaging based on angular spectrum theory. Photoacoustics.

[42] Yang X. (2024). Monte Carlo-based optical simulation of optical distribution in deep brain tissues using sixteen optical sources. Bioengineering.

[43] Menozzi L. (2023). Three-dimensional non-invasive brain imaging of ischemic stroke by integrated photoacoustic, ultrasound and angiographic tomography (PAUSAT). Photoacoustics.

