# Supplementary material for: Water-sensitive photoacoustic temperature characterization at 960 nm in cerebral vascular phantoms with CT co-registration
Source: Photoacoustics. 2026 Jul 21;51:100862. doi: 10.1016/j.pacs.2026.100862 (PMC13425882; doi:10.1016/j.pacs.2026.100862)
Supplement: Supplementary file 1 — S1 Experimental setup and schematic layout [file mmc1.docx]

**Experimental system and schematic layout**

**for photoacoustic thermometry**

**
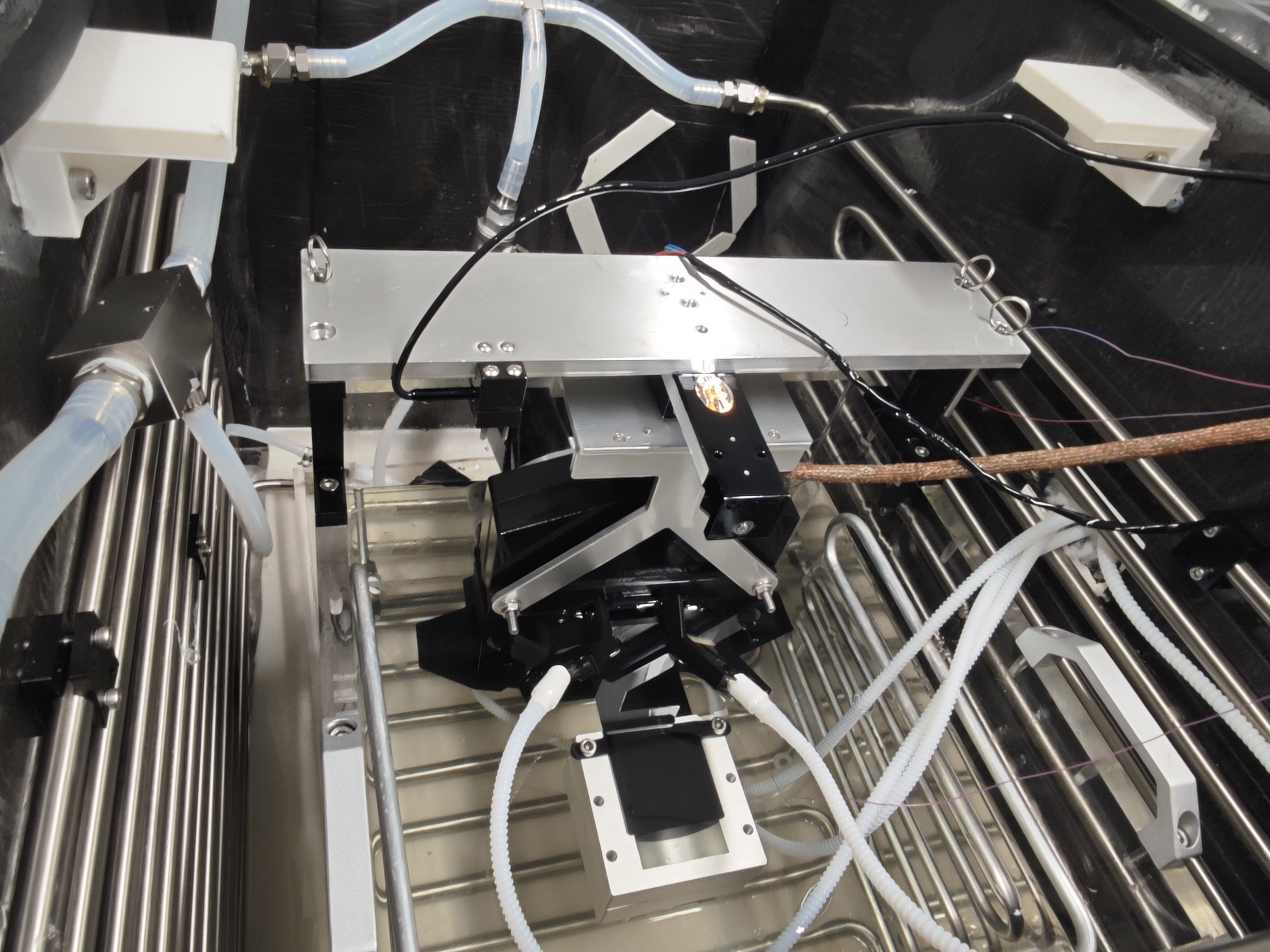
**

**Fig. S1. Photoacoustic thermometry experimental system.**


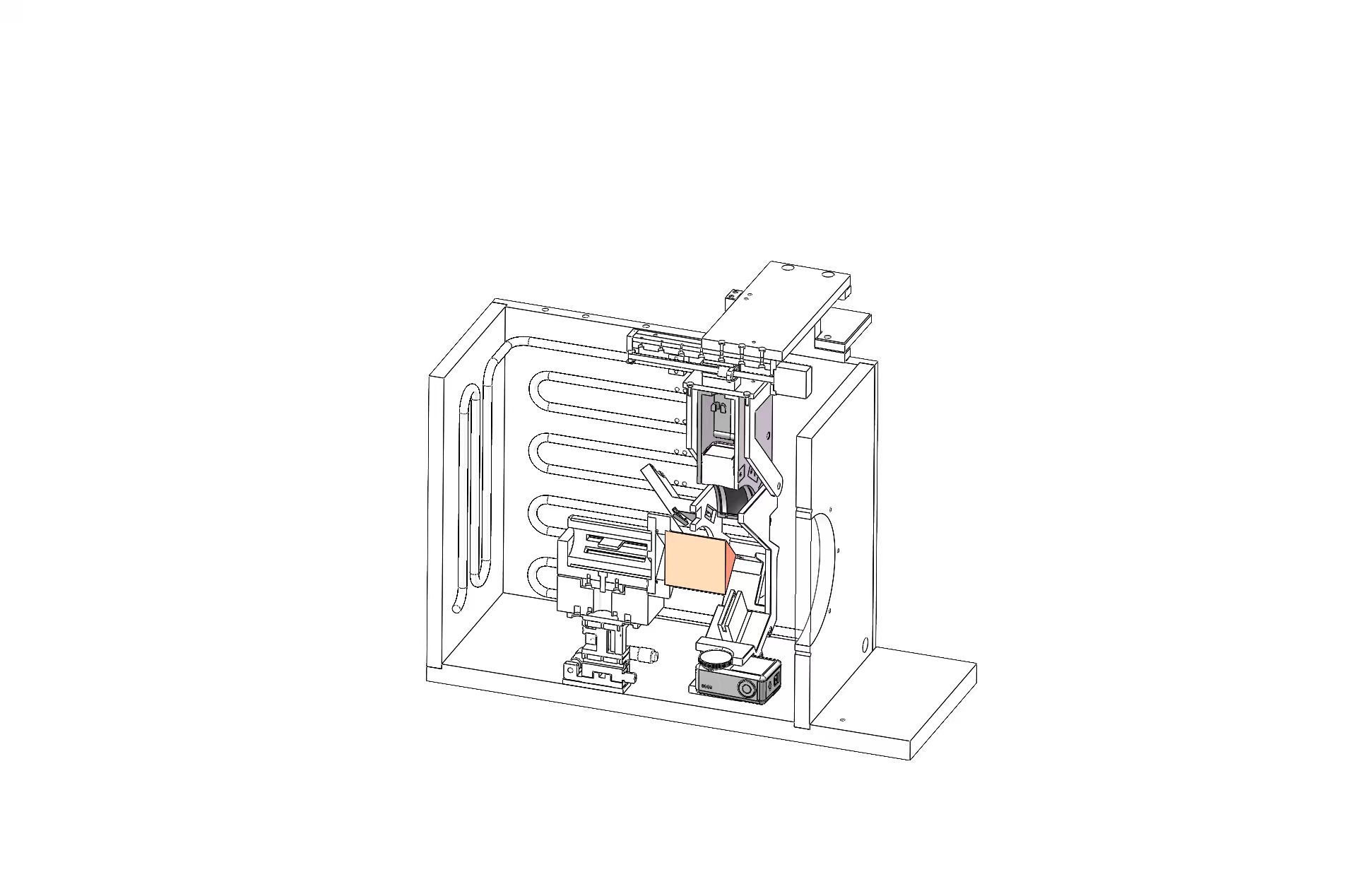


**Fig. S2 Schematic diagram of the photoacoustic thermometry experimental system.** The system consists of a two-stage oil-bath temperature-controlled inner chamber, ethylene glycol circulation pipeline, Y-axis moving platform, ultrasound transducer, three-axis adjustable sample stage, homogenized optical fiber output end, and camera mounting position.
