# Supplementary material for: Water-sensitive photoacoustic temperature characterization at 960 nm in cerebral vascular phantoms with CT co-registration
Source: Photoacoustics. 2026 Jul 21;51:100862. doi: 10.1016/j.pacs.2026.100862 (PMC13425882; doi:10.1016/j.pacs.2026.100862)
Supplement: Supplementary file 2 — S2 Acoustic parameters of the phantom and skull tissue [file mmc2.docx]

**Acoustic parameters of the phantom and skull tissue**

| Material / tissue | Role in this study | Acoustic properties | Density | Key interpretation | Reference |
| --- | --- | --- | --- | --- | --- |
| 1% agarose hydrogel | Water-rich soft-tissue-like background | ~1.49-1.54 km/s | ~1.0g/cm³ | Used as a simplified brain-parenchyma-like matrix, but without the full optical/scattering complexity of real brain tissue | [1, 2] |
| 8001 SLA resin shell | Simplified bone-like high-sound-speed boundary | ~ 3.0 km/s in this study | manufacturer-dependent/not measured | Used only to introduce high-sound-speed acoustic mismatch; not a full skull-equivalent model | [3] |
| Human brain parenchyma | Biological soft-tissue reference | ~1.54 km/s | ~1.04g/cm^3^ | Provides the biological reference for the agarose background | [2, 4] |
| Human skull / cranial bone | Biological transcranial boundary reference | Trabecular bone ~1886m/s; Cortical bone ~3476m/s. | cortical bone ~1.97g/cm^3^ | The SLA shell captures only the high-sound-speed mismatch, not skull layering, attenuation, dispersion, anisotropy, or morphology | [2, 3] |

**Reference:**

1. Menikou, G. and C. Damianou, *Acoustic and thermal characterization of agar based phantoms used for evaluating focused ultrasound exposures.* Journal of therapeutic ultrasound, 2017. **5**(1): p. 14.

2. Culjat, M.O., D. Goldenberg, P. Tewari, and R.S. Singh, *A review of tissue substitutes for ultrasound imaging.* Ultrasound in medicine & biology, 2010. **36**(6): p. 861-873.

3. Pichardo, S., V.W. Sin, and K. Hynynen, *Multi-frequency characterization of the speed of sound and attenuation coefficient for longitudinal transmission of freshly excised human skulls.* Physics in Medicine & Biology, 2011. **56**(1): p. 219-250.

4. Yaroslavsky, A., et al., *Optical properties of selected native and coagulated human brain tissues in vitro in the visible and near infrared spectral range.* Physics in Medicine & Biology, 2002. **47**(12): p. 2059-2073.
