# Supplementary figures and images for: Water-sensitive photoacoustic temperature characterization at 960 nm in cerebral vascular phantoms with CT co-registration
Source: Photoacoustics. 2026 Jul 21;51:100862. doi: 10.1016/j.pacs.2026.100862 (PMC13425882; doi:10.1016/j.pacs.2026.100862)

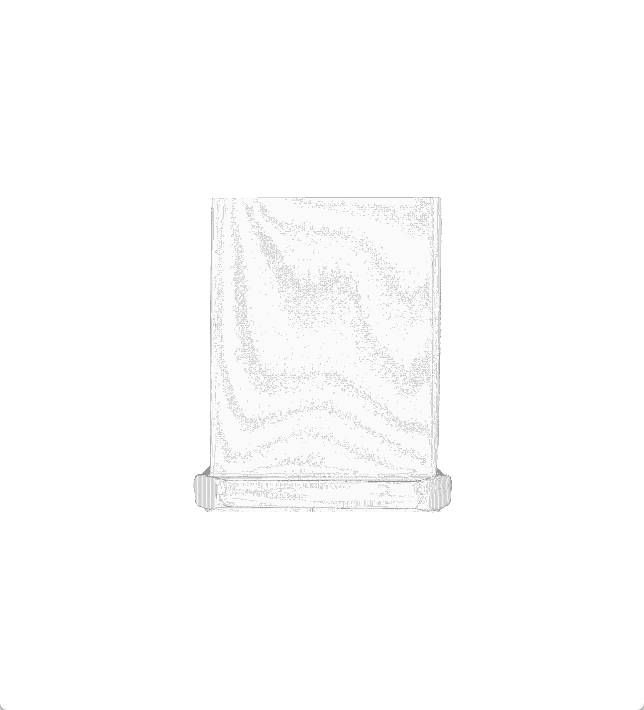

Supplement: Supplementary file 3 — S3 360-degree rotation of CT-scanned shelled phantom [file mmc3.zip › 360-degree_rotation_of_CT-scanned_shelled_phantom.gif]

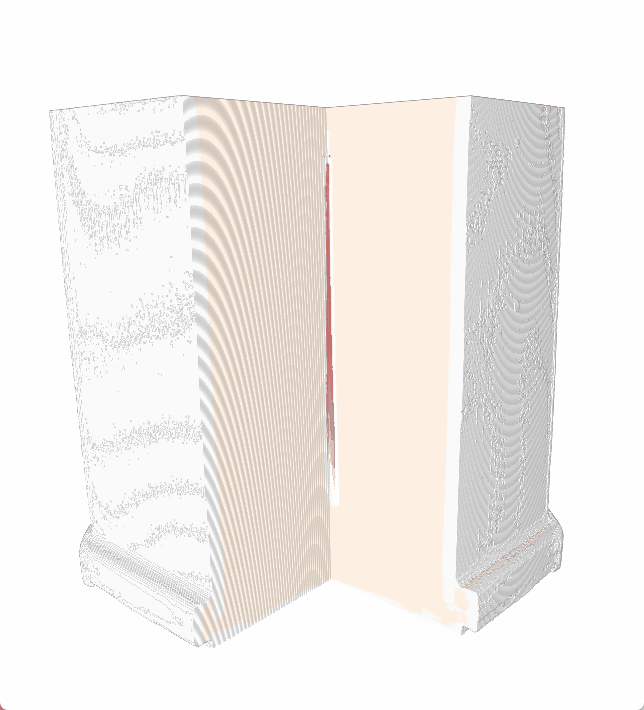

Supplement: Supplementary file 4 — S4 30-degree rotation quarter-sectioned shelled phantom [file mmc4.zip › 30-degree_rotation_quarter-sectioned_shelled_phantom.gif]
